# The Antimalarial Mefloquine Shows Activity against *Mycobacterium abscessus*, Inhibiting Mycolic Acid Metabolism

**DOI:** 10.3390/ijms22168533

**Published:** 2021-08-08

**Authors:** Giulia Degiacomi, Laurent Roberto Chiarelli, Deborah Recchia, Elena Petricci, Beatrice Gianibbi, Ersilia Vita Fiscarelli, Lanfranco Fattorini, Fabrizio Manetti, Maria Rosalia Pasca

**Affiliations:** 1 Department of Biology and Biotechnology “Lazzaro Spallanzani”, University of Pavia, 27100 Pavia, Italy; giulia.degiacomi@unipv.it (G.D.); laurent.chiarelli@unipv.it (L.R.C.); deborah.recchia01@universitadipavia.it (D.R.); 2 Department of Biotechnology, Chemistry and Pharmacy, University of Siena, via Aldo Moro 2, 53100 Siena, Italy; elena.petricci@unisi.it (E.P.); beatrice.gianibbi@student.unisi.it (B.G.); fabrizio.manetti@unisi.it (F.M.); 3 Cystic Fibrosis Diagnostics, Microbiology and Immunology Diagnostics, Bambino Gesù Children’s Hospital IRCCS, 00165 Rome, Italy; evita.fiscarelli@opbg.net; 4 Dipartimento di Malattie Infettive, Istituto Superiore di Sanità, 00161 Rome, Italy; lanfranco.fattorini@iss.it

**Keywords:** *Mycobacterium abscessus*, MmpL3, pharmacophore model, mefloquine, drug repurposing

## Abstract

Some nontuberculous mycobacteria (NTM) are considered opportunistic pathogens. Nevertheless, NTM infections are increasing worldwide, becoming a major public health threat. Furthermore, there is no current specific drugs to treat these infections, and the recommended regimens generally lack efficacy, emphasizing the need for novel antibacterial compounds. In this paper, we focused on the essential mycolic acids transporter MmpL3, which is a well-characterized target of several antimycobacterial agents, to identify new compounds active against *Mycobacterium abscessus* (*Mab*). From the crystal structure of MmpL3 in complex with known inhibitors, through an in silico approach, we developed a pharmacophore that was used as a three-dimensional filter to identify new putative MmpL3 ligands within databases of known drugs. Among the prioritized compounds, mefloquine showed appreciable activity against *Mab* (MIC = 16 μg/mL). The compound was confirmed to interfere with mycolic acids biosynthesis, and proved to also be active against other NTMs, including drug-resistant clinical isolates. Importantly, mefloquine is a well-known antimalarial agent, opening the possibility of repurposing an already approved drug, which is a useful strategy to reduce the time and cost of disclosing novel drug candidates.

## 1. Introduction

Nontuberculous mycobacteria (NTM) pulmonary infections are an emergent global health threat, particularly to individuals that have a predisposing medical condition such as cystic fibrosis (CF), chronic obstructive pulmonary disease (COPD), and bronchiectasis. Over the last two decades, the incidence of NTM infections among CF individuals has raised from 5% to 20%, increasing morbidity and mortality associated with these pathogens [1,2], whereas COPD patients treated with inhaled corticosteroid therapy are associated with a 29-fold increased risk of NTM infections [3]. NTM comprise more than 190 species and subspecies with only a few causing serious and often opportunistic infections in humans. Amongst them, the most commonly identified species in CF individuals are the slow growing *Mycobacterium avium* complex (MAC) and the rapidly growing *Mycobacterium abscessus* complex (MABSC), accounting for 95% of CF cases [4]. MAC mainly consists of *M. avium* subsp. *avium* (*Mav*) and *M. avium* subsp. *intracellulare*, while MABSC includes the following three subspecies: *M. abscessus* subsp. *abscessus* (*Mab*), *M. abscessus* subsp. *bolletii*, and *M. abscessus* subsp. *massiliense*.

A standard of care treatment for NTM is available for only a few pathogens, including MAC and *Mab*. Generally, the therapeutic options consist of multiple antimicrobial agents that are often associated with clinically significant adverse reactions and must be administered for prolonged periods [5]. A multidrug regimen based on a macrolide (clarithromycin or azithromycin) is the recommended treatment for MAC, which also includes rifampicin and ethambutol. Meanwhile, the management of pulmonary infections originated by *Mab* typically consists of an oral macrolide in conjunction with intravenous or inhaled amikacin, and one or more of the following drugs: intravenous cefoxitin, imipenem, or tigecycline, in addition to oral antibiotics (minocycline, clofazimine, moxifloxacin, and linezolid) [6]. The American Thoracic Society and the Infectious Diseases Society of America stated that the optimal drug regimens and duration of anti-*Mab* treatment are not known. Moreover, treatment outcomes are poor, and reinfection with another strain or species is common [5].

The main causes of treatment failure are the intrinsic and acquired antibiotic resistance of NTM in general, and of *Mab* in particular, which is one of the most drug-resistant mycobacteria [7]. Furthermore, the insufficient effectiveness of drugs toward *Mab* is associated with a lack of bactericidal activity of most of the active compounds [3,8]. In this context, there is an urgent need of more active drugs to curb NTM infections.

Nevertheless, de novo drug discovery is a highly costly and time-consuming process [3]. Therefore, the study of approved drugs for new therapeutical applications provides a rapid strategy to clinical implementation.

The drug pipeline for NTM is nearly empty with lead compounds derived either from the repurposing of existing drugs or from the cross-testing of compounds active in tuberculosis [9], but also includes a few new chemical entities. Interestingly, some of the novel drug candidates target MmpL3 (mycobacterial membrane protein Large 3) [10,11,12,13,14]. MmpL3 is an essential inner membrane flippase present in all mycobacteria, which is involved in the last step of mycolic acid biosynthesis. In particular, MmpL3 transports by flipping trehalose monomycolates across the inner membrane into the periplasm, where they are required to form the outer membrane mycolic acids [15,16]. The MmpL3 therapeutic potential has already been unveiled in several studies as a vulnerable and promiscuous target in *Mycobacterium tuberculosis* [4].

In this study, we combined an in silico approach (developing a pharmacophore, from the crystal structure of MmpL3 in complex with the inhibitors AU1235, SQ109, ICA38, and rimonabant [17]) with the repurposing strategy. Our pharmacophore was exploited for the screening of the DrugBank and the National Cancer Institute database, in order to identify novel scaffolds targeting MmpL3 belonging to molecules currently in use, for a future implementation as anti-*Mab* compounds. Amongst them, the disclosure of mefloquine, an antimalarial, was of particular interest.

## 2. Results

### 2.1. Molecular Modeling: Construction of the Pharmacophore

**Figure 1:**
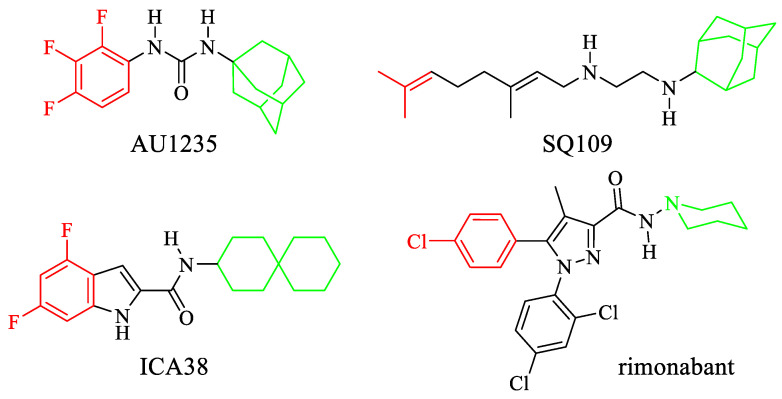
Structure of the MmpL3 inhibitors used to build the pharmacophoric model. The tri-fluorophenyl moiety of AU1235, the difluorophenyl moiety of ICA38, the terminal alkenyl edge of SQ109, and the 5-chlorophenyl moiety of rimonabant are represented in red. The adamantyl moiety of both AU1235 and SQ109, the spiro-undecane moiety of ICA38, and the piperidine ring of rimonabant are represented in green.

The X-ray crystallographic three-dimensional structures of the complexes between MmpL3 and AU1235, SQ109, ICA38, and rimonabant [17] (Figure 1) were used to generate four different pharmacophoric models by means of the software Phase [18].

**Figure 2:**
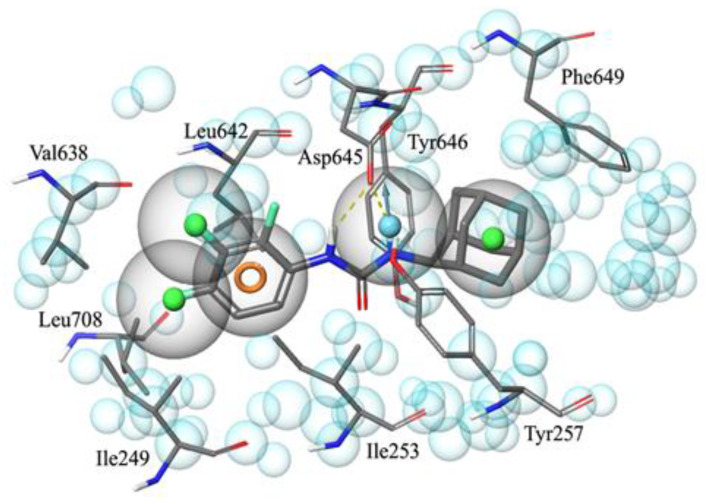
Graphical representation of the final five-feature pharmacophoric model for MmpL3 inhibitors embedded within the binding site that contains AU1235. Amino acids are represented by thin lines (atom type notation), while the inhibitor is rendered by thick lines (atom type notation). Green spheres represent the hydrophobic features of the pharmacophore, the orange circle is the aromatic ring, while the cyan sphere corresponds to the hydrogen bond acceptor feature. Light cyan spheres of variable size represent excluded volumes. Hydrogen bonds between the amidic NH groups of the inhibitor and Asp645 side chain are coded by dashed yellow lines.

Receptor–ligand interactions were coded into automatically generated pharmacophores that also comprised receptor-based excluded volumes. The four pharmacophores were then merged into a common feature pharmacophoric hypothesis constituted by the sole chemical features shared by the parent models. The resulting five-feature pharmacophore shows three hydrophobic regions (represented by green spheres in Figure 2), one aromatic ring (the orange circle), and a hydrogen bond donor (the cyan sphere), in addition to excluded volumes (the transparent cyan spheres of variable size) that represent regions of space occupied by the amino acid side chains and are thus forbidden to portions of the ligand structures. A comparison between the pharmacophore and the MmpL3 binding site showed that the vicinal hydrophobic portions and the aromatic ring feature of the model are accommodated within a pocket (delimited by the side chains of Leu642, Val638, Leu708, Ile249, and Ile253), where the tri-fluorophenyl moiety of AU1235 (the most important molecular portions of the four MmpL3 inhibitors are highlighted in Figure 1), the di-fluorophenyl moiety of ICA38, the 5-chlorophenyl moiety of rimonabant, and the alkenyl edge of SQ109 are found in the corresponding crystallographic complexes. On the other hand, the hydrogen bond acceptor feature of the model takes into account the presence of amide or amine NH groups of the inhibitors that give hydrogen bonds with the side chain of Asp645. The remaining hydrophobic feature of the model represents the adamantyl moiety of both AU12345 and SQ109, as well as the spiro-undecane moiety of ICA38 or the piperidine ring of rimonabant, and it is located in a pocket delimited by Tyr257, Phe649, and Tyr646.

The final pharmacophore was used as a three-dimensional filter to identify 36 putative MmpL3 ligands within databases of known drugs (such as the DrugBank database) [19] and commercially available compounds (such as the National Cancer Institute database) [20] (Table S1).

### 2.2. Characterization of MmpL3 Ligands Active against Mab Growth

**Figure 3:**
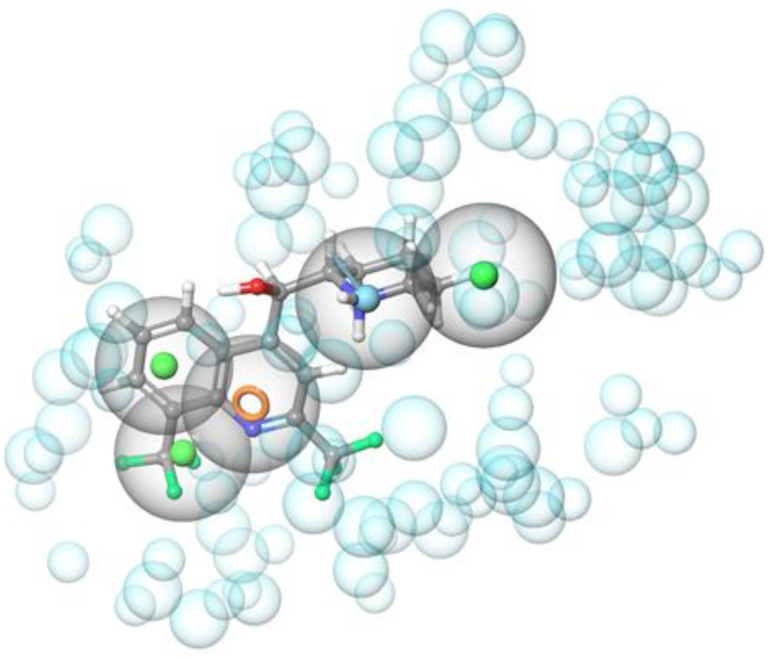
Graphical representation of mefloquine superimposed to the pharmacophoric model. The hydrophobic features (green spheres) are matched by the trifluoromethylphenyl moiety (the most important molecular portions of the mefloquine structure are highlighted in Figure S1) and the alkyl portion of the piperidine ring, the aromatic ring feature (orange circle) is mapped by the pyridine core, and the hydrogen bond donor group (cyan sphere) is represented by the basic piperidine nitrogen atom. Excluded volumes (light cyan spheres of variable size) are also displayed.

As a result, among the 36 prioritized compounds, NSC157387 (mefloquine, **1**), the tricyclic urea NSC120330, and the benzimidazole NSC135792 are active against *Mab* growth with an MIC = 16 μg/mL (Table S1 and Table 1). As an example of a match between the pharmacophore and the chemical portions of the ligands, the trifluoromethylphenyl moiety (the most important molecular portions of mefloquine structure are highlighted in Figure S1) of mefloquine matched the two vicinal hydrophobic regions (Figure 3), while the pyridine ring corresponded to the aromatic ring feature. The basic nitrogen atom in its protonated form was the hydrogen bond donor, and the remaining hydrophobic feature is mapped by the C2-C3 alkyl portion of the piperidine nucleus.

**Figure 4:**
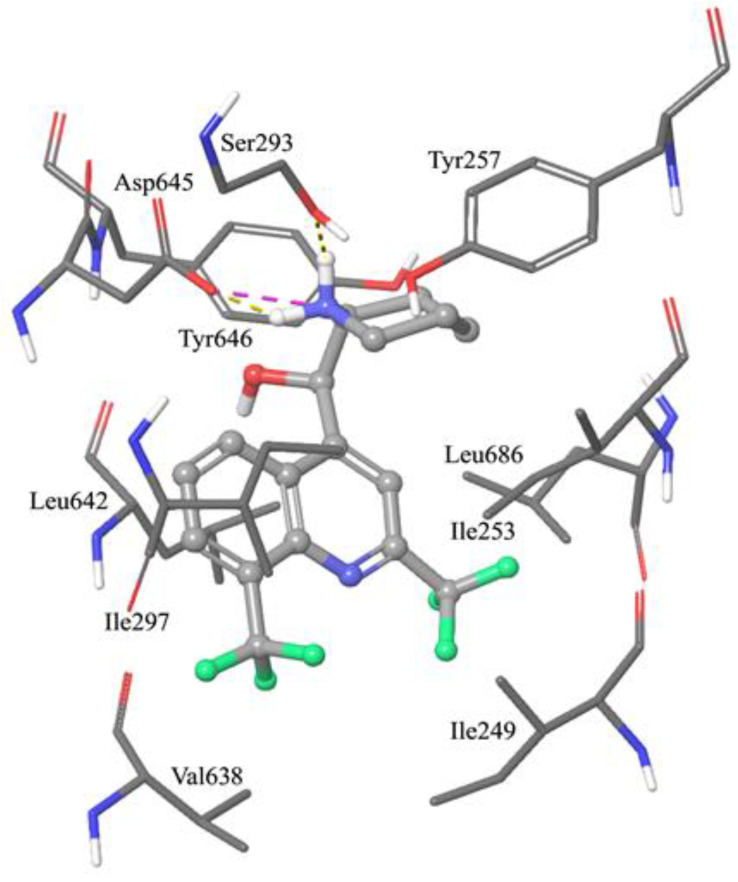
Graphical representation of the interaction pattern of the best-docked pose of mefloquine within the MmpL3 binding site. Extended hydrophobic contacts involve both the trifluoromethyl substituents and the quinoline core. The piperidine basic nitrogen atom makes a hydrogen bond with the Ser293 side chains, and a charge-reinforced hydrogen bond with the carboxyl edge of Asp645. Amino acids are represented by sticks (atom type notation), while mefloquine is represented by a ball and stick (atom type notation). Yellow dashed lines codify for hydrogen bonds, while the magenta dashed line represents a charge-reinforced hydrogen bond.

To further support the hypothesis that such compounds were able to bind MmpL3, they were submitted to molecular docking simulations and minimization of the resulting complexes. An analysis of the best-docked pose of mefloquine within the MmpL3 binding site showed extended hydrophobic contacts between the trifluoromethyl group at C8 and the side chains of Val638 and Ile297 (Figure 4), as well as an additional interaction between the condensed phenyl ring and Leu642. Another hydrophobic pocket comprising Ile249, Ile253, and Leu686 accommodated the 2-trifluromethylpyridyl moiety. Moreover, Ile253 also interacted with the alkyl portion of the piperidine ring, together with Tyr257 and Tyr646. The most important anchor point of mefloquine was represented by the basic nitrogen atom of the piperidine nucleus that was able to make a hydrogen bond with the side chain of Ser293 and an additional charge-reinforced hydrogen bond with the carboxylic edge of Asp645, found to play a pivotal role for the interaction of MmpL3 with all the co-crystallized ligands. A bi-dimensional diagram of the interactions between mefloquine and amino acid residues mostly contributing to the stabilization of the MmpL3-mefloquine complex, and distances between molecular portions of mefloquine and amino acid groups are depicted in Figure S2.

### 2.3. SAR Considerations of the 3 Anti-Mab MmpL3 Ligands

A SAR analysis on a small library of an additional 12 mefloquine derivatives (**2–13**) provided by the National Cancer Institute suggested that the basic piperidine ring (the black portion in Figure S1) at the appropriate distance from the quinoline nucleus (the green portion in Figure S1) was mandatory for its activity against *Mab* wild-type strain (Table 1).

In fact, the opening of the piperidine ring (**3**–**7**) or lengthening of the linker between the two cycles (**2**) resulted in the loss of antimycobacterial activity. On the other hand, the replacement of the C8 trifluoromethyl moiety with a chloride maintained activity only if the piperidine ring was kept intact. In fact, **9** and **11** showed a MIC of 16 and 32 μg/mL, respectively, while **10** was inactive. Moreover, further reductions in the size and hydrophobic character of the C8 substituent to F and H led to inactive compounds (**8** and **12**). Finally, the condensed derivative **13** showed an interesting antimycobacterial activity of 16 μg/mL. The binding pose and the interaction pattern hypothesized for mefloquine agreed with the major SAR found for its congeners. In fact, mefloquine derivatives with a linear or branched amine side chain (**3**–**7**) underwent conformational rearrangements that led to the lack of the pivotal hydrogen bonds with Asp645 and Ser293. Moreover, a significant reduction in the size and lipophilicity of the C8 substituent to F and H nullified the hydrophobic interactions found for the CF_3_ group of mefloquine and affected negatively the antimycobacterial activity. 

A small library of an additional 8 compounds provided by Enamine Ltd. (Kyiv, Ukraine) was also built around the tricyclic urea NSC120330; however, none of the assayed compounds were active (Table S2). This SAR trend could be reasonably justified by the structural properties of such compounds. In fact, NSC120330 showed the presence of an alkylating edge that lacked in the congeneric compounds. Consequently, we can assume that the antimycobacterial activity of NSC120330 was not target-specific and was derived from its alkylating properties. On this basis, NSC120330 and its analogs were not further studied. 

### 2.4. Analysis of Lipid Composition of Mab Cells 

To confirm that mefloquine and NSC135792 actually target MmpL3, we analyzed the global lipid profile and the mycolic acids content of *Mab* cells, upon treatment with both compounds. To this purpose, *Mab* cells were grown in the presence of 25× MIC of the compounds, harvested after 48 h of incubation, and the lipids were extracted and analyzed as reported in Material and Methods. As a positive control, cells were grown in the presence of 10X MIC of the well-known MmpL3 inhibitor BM212 [21]. 

**Figure 5:**
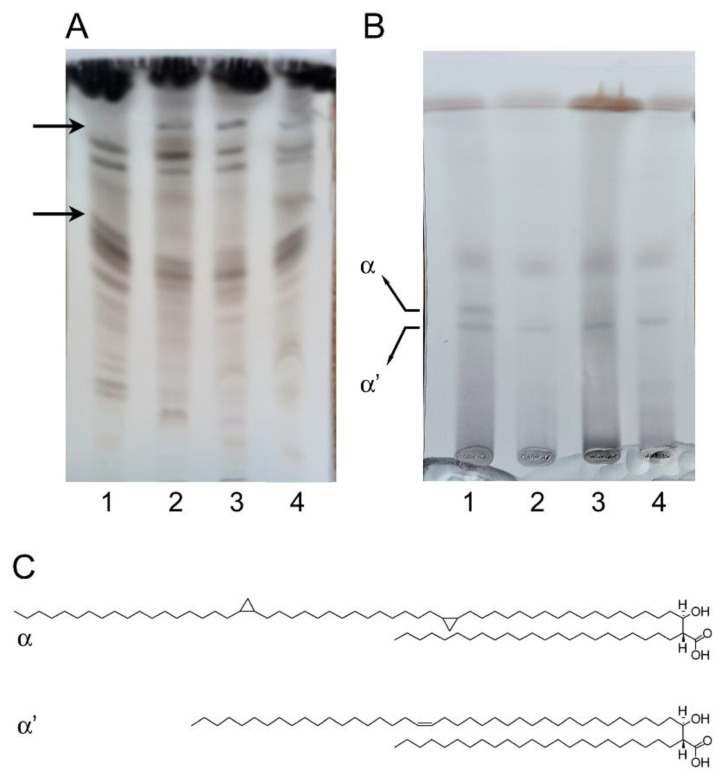
Effects of the compounds on *Mab* cell wall composition. *Mab* cells were grown for 48 h in the absence/presence of compounds, and the lipid composition was then analyzed as in Material and Methods. (**A**) TLC analysis of the complete lipid profile extracted and separated in solvent CHCl_3_/CH_3_OH/H_2_O (20:4:0.5). (**B**) total mycolic acid methyl esters (MAMEs) and fatty acid methyl esters (FAME) extracted from delipidated cell pellets were separated in hexane/ethyl acetate (95:5). α and α’-mycolic acids correspond to long-chain (C77–79) and short-chain (C62–64) mycolic acids in *Mab* [10]. Lane 1: no addition; lane 2: 10× MIC BM212; lane 3: 25× MIC NSC135792; lane 4: 25× MIC mefloquine (4). Figures are representative of three independent experiments. (**C**) Structures of α and α’-mycolic acids [23].

As shown in Figure 5, the global lipid profiles of *Mab* cells treated with both mefloquine and NSC135792 were very similar to those of the cells treated with BM212 (positive control).

*Mab* cultures grown in the presence of the higher solvent concentration used (4% DMSO for mefloquine and NSC135792, 4% ethanol for BM212) as a negative control did not show significant differences with untreated cultures.

It is known that in *M. tuberculosis*, BM212 acts to block the TDM transport by inhibiting MmpL3 [22]. Indeed, as it was nicely demonstrated by Xu et al., 2017 [21], its mechanism of action is not due to indirect effects on the proton motive force, but BM212 binds MmpL3, directly inhibiting its activity. 

In our cases, cells treated with NSC135792 and mefloquine, as well as with BM212, showed significant changes in the composition of the mycobacterial cell envelope in comparison with the untreated control, with at least two main spots altered, as indicated by the arrows in Figure 5A.

It was demonstrated that the effects of the tested compounds were concentration-dependent, being negligible at 1×-MIC concentration and more visible at 10×- and 25×-MIC concentrations (Figure S3). A comparable effect on lipid profile was found with BM212, suggesting that NSC135792 and mefloquine likely act through the inhibition of MmpL3 activity, although the mechanism of action of the pyrrole derivative has been demonstrated in *M. tuberculosis*, but not in *Mab* yet.

Furthermore, mycolic acids were also extracted from delipidated bacteria to observe the possible impact on arabinogalactan (AG) mycolylation. The presence of both compounds led to a very significant decrease in α-mycolates (Figure 5B), as also visible in the positive control sample (lane 2). The decrease we observed could be due to the block of TMM transport that could indirectly perturb AG mycolylation in all the treated samples. 

It is conceivable that TMM transport could be actually blocked by the studied compounds, as well as BM212 in *M. tuberculosis*. On the other hand, we cannot exclude that the observed phenotype could be the result of a possible inhibition in different steps of mycolic acids biosynthesis, specifically of the latter condensation steps catalyzed by the FAS-II system. In fact, the compounds under investigation affected only one form of mycolates, the α-mycolates (Figure 5C).

Nevertheless, the two compounds (mefloquine and NSC135792) behave overall in a similar way with respect to the control BM212, suggesting that they possibly share the same mechanism of action; however, at this stage of the study, we can assume that an inhibition in different steps of mycolic acids biosynthesis occur, causing the observed phenotype.

### 2.5. Evaluation of Antimycobacterial Activity of Selected Compounds against NTM Clinical Isolates

We assessed the sensitivity to mefloquine and NSC135792 against a panel of both reference strains and drug-resistant clinical isolates belonging to MABSC and MAC. The micro-broth dilution method was used to determine the MIC values.

We tested, in addition to *M. abscessus* ATCC 19977, *M. bolletii* 1999-0888 and *M. massiliense* 2005-0524 (MABSC), alongside five *Mab* clinical isolates resistant to at least a quinolone and a tetracycline, and two *Mab* multidrug-resistant (MDR) isolates, one of them isolated from a CF patient. Moreover, *M. avium* subsp. *avium* Chester ATCC15769 reference strains (MAC) and four *M. avium* isolates resistant to at least linezolid were used (Table 2).

Interestingly, mefloquine and NSC135792 were also active against other NTM strains and NTM drug-resistant clinical isolates, also including *Mab* MDR strains.

### 2.6. Drug Combination with Mefloquine against Mab

The potential inclusion of mefloquine in combinational *Mab* therapy was studied via Checkerboard Assay (CBA). NSC135792 was not further investigated, because the disclosure of a 35-compound library constituted 5,6-disubstituted-benzimidazole analogs with antimycobacterial activity as low as 0.125 μg/mL toward *Mab* (CIP104536) [24]. Bedaquiline, ciprofloxacin, and clarithromycin were tested. The main indicator of synergy, the Fractional Inhibitory Concentration Index (FICI), was >0.5–4 for each assayed antibiotic, indicating no antagonism interaction with mefloquine and, thus, the suitability of mefloquine for a possible combinational therapy.

## 3. Discussion

Despite the increasing incidence in NTM infections, currently, there are no specific drugs against them. Moreover, the recommended regimens for the treatment of *Mab* lung infections generally lack efficacy [25], emphasizing the urgent need for new antibacterial compounds with novel mechanisms of action, also considering that very few molecules are in clinical development [8]. 

The cell wall biosynthesis pathways have represented a rich source of validated targets for antimycobacterial compounds. *Mab* does not represent an exception, as the majority of new compounds under investigation for its treatment are mycolic acid biosynthesis inhibitors. Among these targets, MmpL3 has emerged as the target of several different chemical entities showing antitubercular activity, being defined as a promiscuous target [26]. Interestingly, several of these inhibitors have been found active also against *Mab* or *M. avium* [4,14,27]. Moreover, novel *Mab* active compounds targeting MmpL3 have been identified through the phenotypic screen of compound libraries [10,28].

In this context, our study aimed to identify novel scaffolds, already commercially available, inhibiting MmpL3, that will be useful for the development of new anti-*Mab* compounds. To this purpose, we used an in silico approach, developing a pharmacophore, from the crystal structure of MmpL3 in complex with the antitubercular inhibitors AU1235, SQ109, ICA38, and rimonabant [17].

The screening of the DrugBank database and the National Cancer Institute database exploiting our pharmacophore afforded 36 prioritized compounds. Three of them (namely, mefloquine, the tricyclic urea NSC120330, and the benzimidazole NSC135792) showed an interesting activity against *Mab* growth (MIC = 16 μg/mL). In particular, we confirmed that mefloquine and NSC135792 interfere with mycolic acid biosynthesis, perturbing indirectly the AG mycolylation (Figure 5B). It is plausible that the two compounds under investigation could share the same mechanism of action of the control and well-known MmpL3 inhibitor BM212, but it is not possible to exclude an inhibition downstream MmpL3, as a reduction of only α-mycolates was observed. Meanwhile, the indole-2-carboxamides and the piperidinol derivative PIPD1, two anti-*Mab* MmpL3 inhibitors, affect the formation of both α and α’ mycolates [8,9]. 

Interestingly, some analogs of NSC135792 have been recently published as very active compounds against *Mab*, inhibiting MmpL3 activity [23]. For this reason, this compound was not further investigated; nevertheless, this evidence supports the validity of our pharmacophore model.

Another compound that emerged from our in silico analysis is the mefloquine, which is a well-known antimalarial compound [29]. This molecule is particularly interesting, opening the possibility of a drug repurposing strategy, an approach particularly considered for the antibacterials. Indeed, the emergence of antibiotic-resistance often overtakes the development of novel drugs, and in this context, the repurposing of already approved ones can be useful to reduce time and cost [8]. The characterization of the antimicrobial spectrum of mefloquine demonstrated that the compound is significantly active against other MABSC subspecies, including multidrug-resistant clinical isolates, and also displayed moderate activity against different MAC strains. Moreover, the compound did not show antagonistic effects in combination with other antimycobacterial drugs, as demonstrated by CBA, confirming that it could be used for the development of new drug combinations.

In conclusion, with our study, we developed a pharmacophore model that allowed one to disclose novel scaffolds inhibiting the mycolic acids biosynthesis, endowed with significant anti-*Mab* activity. Furthermore, the identification of mefloquine among these compounds will allow further investigation for a repurposing strategy to disclose novel drug candidates.

## 4. Materials and Methods

### 4.1. Computational Details

Small molecules were sketched or imported in SMILES notation in the Maestro suite [30] and were prepared with the LigPrep routine, generating possible tautomers at pH 7 ± 1 with Epik [31] and using the OPLS3e force field. The X-ray crystallographic three-dimensional structure of MmpL3 in complex with AU1235 (entry 6ajh of the protein data bank, 2.8 Å resolution), SQ109 (entry 6ajg, 2.6 Å resolution), ICA38 (entry 6ajj, 2.8 Å resolution), and rimonabant (entry 6aji, 2.9 Å resolution) were imported from the protein data bank and submitted to the Protein Preparation Wizard by assigning bond orders and adding hydrogens to the appropriate positions. Next, starting from each receptor–ligand complex, a pharmacophore model was generated by means of the Develop Pharmacophore Model routine within the software Phase, adding a receptor-based excluded volume shell. The Hypothesis Alignment was then applied to finding feature matching between the four original pharmacophores, and only five pharmacophoric features shared by all the models were kept in the final pharmacophoric model. The latter was constituted by three hydrophobic regions, an aromatic ring feature, and a hydrogen bond donor group. The pharmacophore was used as a three-dimensional query to filter both the DrugBank (a comprehensive, free-of-access, online database of about 14,000 entries, including small-molecule drugs, nutraceuticals, and experimental drugs) and the NCI/DTP Open Chemicals Repository (a collection of more than 200,000 compounds provided at no cost upon request). Twenty-eight small molecules were prioritized as putative MmpL3 inhibitors and submitted to biological evaluation. Three of them, namely, mefloquine (NSC157387), the arylurea NSC120330, and the 2-amino-5,6-dimethylbenzimidazole NSC135792, showed an antimycobacterial MIC of 16 μg/mL. In the attempt to find more active compounds and to enlarge SAR considerations, twelve additional mefloquine analogs were collected from the NCI Repository. On the contrary, the arylurea derivatives were abandoned as a consequence of the hypothesis that the activity of NSC120330 (a chloroethylurea) was based on its alkylating properties. In a similar way, the benzimidazole class was not further studied, because its members were already reported as inhibitors of *M. abscessus* [23].

To further support the hypothesis that mefloquine could represent a MmpL3 ligand, molecular docking simulations were also performed by means of the software Glide [32]. In particular, the Receptor Grid Generation module was applied to codify the physico-chemical properties of the MmpL3 binding site to be used during docking calculations. The grid box center (x, y, and z coordinates: 35.13, 3.69, −20.55) was embedded in a 15 × 15 × 15 Å inner box and a 35 × 35 × 35 Å outer box. All the receptor hydroxyl and thiol groups within the receptor grid were allowed to rotate. Next, a standard precision calculation with flexible ligand sampling was set with post-docking minimization of the resulting complexes.

### 4.2. Bacterial Strains, Culture Media, and Chemicals

*Mab* ATCC 19977 and *Mycobacterium avium* subsp. *avium* Chester (ATCC15769) reference strains were used alongside a series of clinical isolates belonging to *Mab* complex (MABSC) and *M. avium* complex (MAC) (Table 2). These strains were grown at 37 °C in Middlebrook 7H9 broth (Difco, Becton Dickinson, NJ, USA), supplemented with 0.05% *w*/*v* Tween 80 or on Middlebrook 7H11 (Difco, Becton Dickinson, NJ, USA), both supplemented with 0.2% *w*/*v* glycerol and 10% *v*/*v* Middlebrook OADC enrichment (oleic acid, albumin, d-glucose, catalase; Becton Dickinson, NJ, USA). 

*Mab* MDR clinical strain n. 1 was isolated at the Centre hospitalier universitaire Vaudois (CHUV, Lausanne, Switzerland) and its antibiotic resistance profile was obtained by the resazurin-based microtiter assay (REMA).

*Mab* MDR clinical isolate n. 2 was identified from a cystic fibrosis patient at the Bambino Gesù Children’s Hospital (Rome, Italy); the sensitivity to antibiotics was detected by the microbroth dilution method (RAPMYCO, Thermo Fisher Scientific, Waltham, MA, USA).

The drug-resistant *Mab* and *M. avium* clinical strains were isolated at the Istituto Superiore di Sanità (Rome, Italy). The MIC values were determined by the Sensititre tests SLOMYCOI for *M. avium* e RAPMYCOI for *M. abscessus*.

All the tested compounds were dissolved in DMSO (Sigma Aldrich, St. Louis, MO, USA). BM212 (Cayman Chemical, Ann Arbor, MI, USA) was dissolved in ethanol 70%. 

### 4.3. MIC Determination

Drug susceptibility of *Mab* was determined using the REMA method [33]. Log-phase cultures were diluted at concentrations of approximately 10^6^ bacteria/mL. Then, 100 μL of the bacterial suspensions was added in a 96-well black plate (Fluoronunc, Thermo Fisher, Waltham, MA, USA) containing 100 μL of Middlebrook 7H9, without the addition of Tween 80, in the presence of serial compound dilution. Growth controls containing no compound and sterile controls without inoculum were also included. A volume of 10 μL of resazurin (0.025% *w*/*v*) was added to each well after 24 h, and bacterial viability was assessed after a further 18–24 h of incubation using a FluoroskanTM Microplate Fluorometer (Thermo Fisher Scientific, Waltham, MA, USA; excitation = 544 nm, emission = 590 nm). Bacterial viability was calculated as a percentage of resazurin turnover in the absence of compound.

MICs of the clinical isolates were determined by means of the micro-broth dilution method. Dilutions of clinical isolates (about 10^6^ CFU/mL) were streaked onto 7H11 solid medium containing a range of drug concentrations. Plates were incubated at 37 °C for about 5 days for MABSC or 7 days for MAC. The growth was visually evaluated: the lowest drug dilution at which visible growth failed to occur was taken as the MIC value. Results were expressed as the average of at least three independent replicates.

### 4.4. Analysis of the Lipid Composition of Mab Cells

The analysis of lipid composition was performed by adapting the procedure previously reported for *M. tuberculosis* [16].

Briefly, *Mab* cultures were grown in Middlebrook 7H9 under standing conditions at 37 °C. After 24 h, mefloquine and NSC135792 were added to the cultures at a concentration of 25-fold MIC for 48 h. BM212, a known inhibitor of MmpL3, was used as a positive control. Then, cells were harvested by centrifugation and pellets were used for analysis. Cells were delipidated by extractions with 6 mL of chloroform/methanol (1:2) for 2 h, stirring at 56 °C, followed by two extractions with 6 mL of chloroform/methanol (2:1). Extracted lipids were subjected to biphasic washes with chloroform/methanol/water (4:2:1), dried under vacuum. Residues were resuspended in chloroform/methanol (2:1), in a volume of 100 μL for 30 mL of culture with an OD_600_ of 0.4. Samples (10 μL) were loaded on a TLC plate and developed in chloroform/methanol/water (20:4:0.5). Lipids were visualized with CuSO_4_ (10% in 8% phosphoric acid) and heating. Pellets from the same cultures were used to analyze the cell wall-bound mycolic acids. To this purpose, delipidated cell pellets were subjected to alkali saponification with 2 mL of 15% tetrabutylammonium hydroxide solution (TBAH) at 100 °C overnight. After cooling, 3 mL of dichloromethane, 2 mL of water, and 300 μL of iodomethane were added and stirred at rt, for 4 h. The mixtures were then centrifuged, aqueous phases were removed, and the organic phases were washed with water and dried under vacuum. For the extraction of mycolic acid methyl esters (MAMEs), samples were resuspended in 3 mL of diethyl-ether, sonicated, and after centrifugation, dried under vacuum. Extracts were then resuspended in chloroform/methanol (2:1), according to the OD_600_ of the culture as above, and 20 μL was loaded on TLC, developed in *n*-hexane/ethyl acetate (95:5, 3-fold), and visualized with CuSO_4_.

### 4.5. Checkerboard Assay

To determine pairwise drug interactions, we used CBA assays. Briefly, serial dilutions of drug partners were performed at different combination ratios in a 96-well black plate (Fluoronunc, Thermo Fisher, Waltham, MA, USA); then, 100 μL of a bacterial inoculum, approximately of 10^6^ CFU/mL, was added. Growth controls containing no compound and sterile controls without inoculum were also included. After 24 h of incubation at 37 °C, 10 μL of resazurin (0.025% *w*/*v*) was added to each well. The effect of the combination was measured using growth inhibition as the endpoint readout after a further 18–24 h of incubation, by means of a FluoroskanTM Microplate Fluorometer (Thermo Fisher Scientific, Waltham, MA, USA; excitation = 544 nm, emission = 590 nm). Then, the FICI value was calculated. The MIC of drug A in the presence of drug B divided by the MIC of drug A alone (FIC_A_ = [MIC_A(B)_/MIC_A_]) is defined as the FIC of drug A (FIC_A_); and vice versa (FIC_B_ = [MIC_B(A)_/MIC_B_]). The sum of these values gives the final parameter FICI. “Synergy” is defined as a ≥4-fold reduction in the MICs of both compounds in combination compared to their MICs alone (FICI ≤ 0.5); “no interaction” when the MIC of one of the compounds remained in the range of ½× to 4× MIC (FICI > 0.5–4); and “antagonism” when the MIC of both compounds is, at least, 4-fold higher than that compared to the activity of the compounds alone (FICI > 4.0) [34].

## Figures and Tables

**Table 1:** Structure and anti-*Mab* activity of 3 active MmpL3 ligands and their analogs.

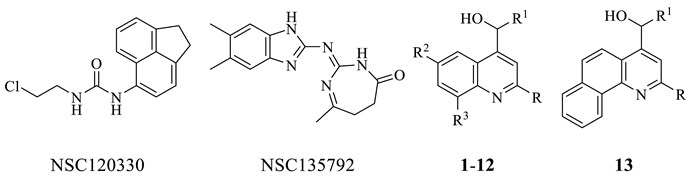					
Compound	R	R^1^	R^2^	R^3^	MIC Against *M. abscessus* ATCC 19977 (μg/mL) ^a^
NSC120330					16
NSC135792					16
NSC157387, mefloquine, **1**	CF_3_	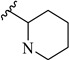	H	CF_3_	16
NSC305803, **2**	CF_3_	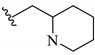	H	CF_3_	>64
NSC305814, **3**	CF_3_	-CH_2_NH(CH_2_)_2_CH_3_	H	CF_3_	>64
NSC305816, **4**	CF_3_	-CH_2_N(CH_2_CH_2_CH_3_)_2_	H	CF_3_	>64
NSC305815, **5**	CF_3_	-CH_2_NH C(CH_3_)_3_	H	CF_3_	>64
NSC305817, **6**	CF_3_	-(CH_2_)_2_NH(CH_2_)_2_CH_3_	H	CF_3_	>64
NSC305793, **7**	CF_3_	-(CH_2_)_2_N((CH_2_)_3_CH_3_)_2_	H	CF_3_	32
NSC158680, **8**	CF_3_	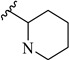	H	F	>64
NSC4377, **9**	*p*-Cl-Phenyl	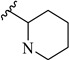	H	Cl	16
NSC369051, **10**	*p*-Cl-Phenyl	-CH_2_N((CH_2_)_3_CH_3_)_2_	Cl	Cl	>64
NSC305758, **11**	Phenyl	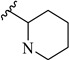	Cl	Cl	32
NSC13446, **12**	cyclohexyl	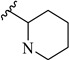	H	H	>64
NSC13480, **13**	Phenyl	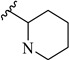			16

^a^ Data representative of three independent experiments.

**Table 2:** Antimycobacterial activity of mefloquine and NSC135792 against NTM reference strains and drug-resistant clinical isolates.

Strains	Genotype	MIC (μg/mL)		Provenience
		Mefloquine	NSC135792	
*M. abscessus* ATCC 19977	Wild-type	16	16	Reference strain
*M. bolletii* 1999-0888	Wild-type	32	32	Hôpital Raymond-Poincaré (Paris, France)
*M. massiliense* 2005-0524	Wild-type	32	32	
*M. abscessus* MDR clinical isolate 1	Resistant to AMK, CLR, DOX, BDQ, CPFX, ERY, MEM, ECO, EMB, ETH, LAN, PRI, RIF, RIP, SQ109, SZD, TAC	32	32	Centre hospitalier universitaire vaudois (Lausanne, Switzerland)
*M. abscessus* MDR clinical isolate 2 (from CF patient)	Resistant to AMK, AMOX, CL, CFP, CFX, CTX, CPFX, DOX, IPM, LZD, MIN, MFX, TOB, TMP-SMX	32	32	Ospedale Pediatrico Bambino Gesù (Rome, Italy)
*M. abscessus* clinical isolate n. 6	Resistant to CLR, MFX, DOX, LZD	32	32	ISS, Rome, Italy
*M. abscessus* clinical isolate n. 7	Resistant to CLR, AMK, MFX, DOX, (Intermediate sensitivity to LZD)	32	32	ISS, Rome, Italy
*M. abscessus* clinical isolate n. 8	Resistant to MFX, DOX	32	32	ISS, Rome, Italy
*M. abscessus* clinical isolate n. 9	Resistant to CLR, MOX, DOX	32	32	ISS, Rome, Italy
*M. abscessus* clinical isolate n.10	Resistant to MOX, DOX	32	32	ISS, Rome, Italy
*M. avium* subsp. avium Chester ATCC15769	Wild-type	64	64	Reference strain
*M. avium* clinical isolate n.1	Resistant to LZD (Intermediate sensitivity to MOX)	64	64	ISS, Rome, Italy
*M. avium* clinical isolate n.2	Resistant to LZD (Intermediate sensitivity to MOX)	64	64	ISS, Rome, Italy
*M. avium* clinical isolate n.3	Resistant to LZD (Intermediate sensitivity to MOX)	64	64	ISS, Rome, Italy
*M. avium* clinical isolate n.4	Resistant to LZD and MOX	64	64	ISS, Rome, Italy

Abbreviations: AMK amikacin, AMOX amoxicillin, BDQ bedaquiline, CL clavulanic acid, CFP cefepime, CFX cefoxitin, CLR clarithromycin, CTX ceftriaxone, CPFX ciprofloxacin, DOX doxycycline, ECO econazole, EMB ethambutol, ERY erythromycin, ETH ethionamide, IPM imipenem, LAN lansoprazole, LZD linezolid, MEM meropenem, MIN minocycline, MFX moxifloxacin, PRI pristinamycin, RIF rifampicin, RIP rifapentine, SQ109, SZD sutezolid, TAC thiacetazone, TMP-SMX trimethoprim/sulfam, TOB tobramycin.

## Supplementary Materials

Supplementary Materials are available online at https://www.mdpi.com/article/10.3390/ijms22168533/s1.
