# Supplementary material for: The Antimalarial Mefloquine Shows Activity against Mycobacterium abscessus, Inhibiting Mycolic Acid Metabolism
Source: Int J Mol Sci. 2021 Aug 8;22(16):8533. doi: 10.3390/ijms22168533 (PMC8395226; doi:10.3390/ijms22168533)
Supplement: Supplementary file 1 [file ijms-22-08533-s001.zip › ijms-1318662-supplementary.pdf]

International Journal of
Molecular Sciences

MDPI

Table S1. Antimycobacterial activity (obtained by REMA) of 36 MmpL3 inhibitors selected *in silico* from the DrugBank. Database and the NCI database (NSC compounds).

Compound	MIC of *M. abscessus* ATCC 19977 (μg/mL)[a]
TAK-242	>64
Efinaconazole	>64
Mefloquine hydrochloride, NSC157387	16
Omeprazole	>64
Triclabendazole	64
Cloxacillin sodium salt	>64
Nizatidine	>64
Cimedidine	>64
NSC13090	>64
NSC43930	>64
NSC47684	>64
NSC52079	64
NSC55233	>64
NSC61717	>64
NSC64876	>64
NSC95470	>64
NSC114866	>64
NSC119922	>64
NSC120330	16
NSC135792	16
NSC142015	>64
NSC154751	>64
NSC164513	>64
NSC168637	>64
NSC190360	>64
NSC204267	>64
NSC205465	>64
NSC303252	>64
NSC339676	>64
NSC354213	>64
NSC369051	>64
NSC371861	>64
NSC379875	>64
NSC608858	>64
NSC618783	>64
NSC621752	>64
Ciprofloxacin	2-4

[a]Data representative of three independent experiments.

Table S2. Structure and activity data of 8 arylurea analogues of NSC120330 provided by Enamine Ltd.

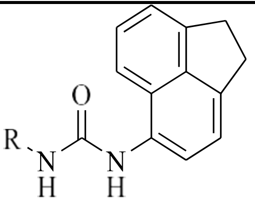

Compound	R	MIC of *M. abscessus* ATCC 19977 (μg/mL)
NSC120330	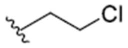	16
Z1138097328	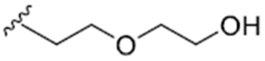	>64
Z1186987371	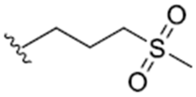	>64
Z1011319648	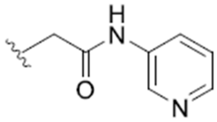	>64
Z2003320996	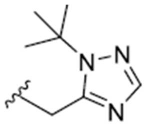	>64
Z1622916397	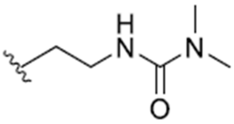	>64
Z1616234919	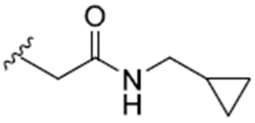	>64
Z1736587836	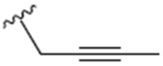	>64
Z813318852	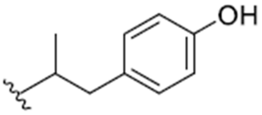	64

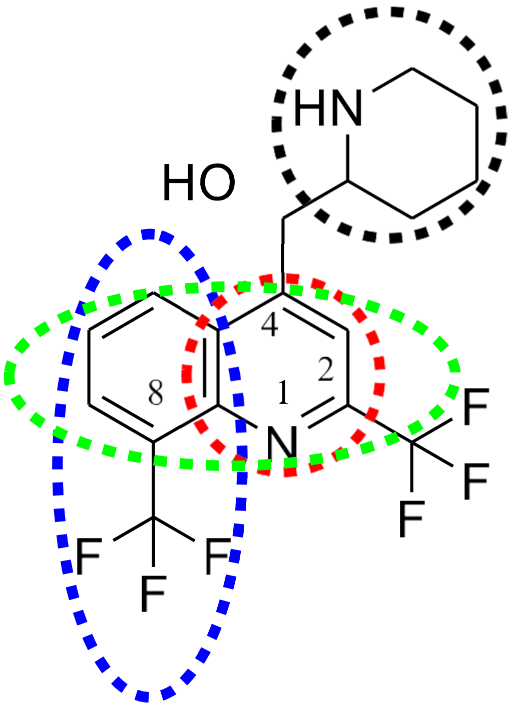

Figure S1. Schematic representation of the major chemical portions important for the interactions of mefloquine with amino acid residues of the binding site within MmpL3. The trifluoromethylphenyl moiety, the pyridine nucleus, the quinoline core, and the piperidine terminus are embedded within the blue, red, green, and black dotted lines, respectively. Several atoms of the quinoline core are numbered to facilitate identification of substituents.

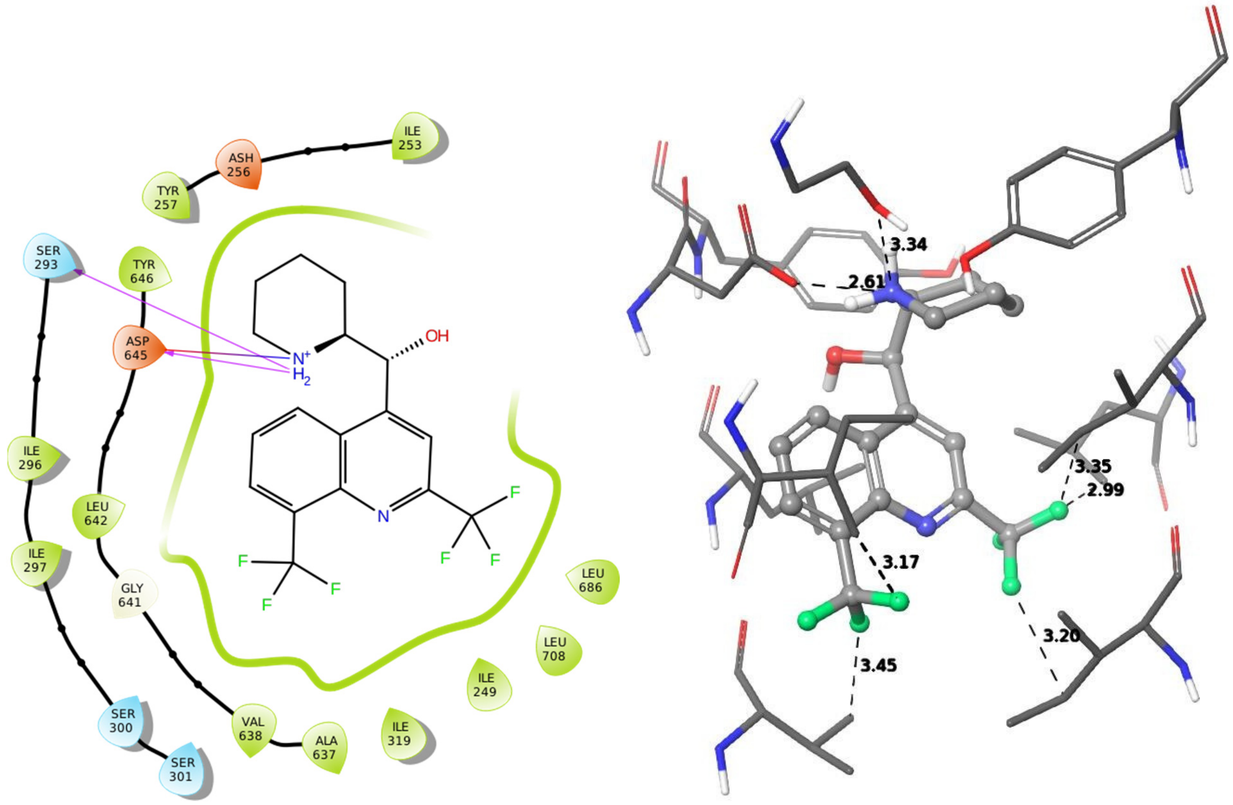

Figure S2. Schematic representation of interactions between mefloquine and the binding pocket within MmpL3. Bi-dimensional diagram of the interactions between mefloquine and amino acid residues mostly contributing to the stabilization of the MmpL3-mefloquine complex (left), and distances (shown by black dashed lines with the corresponding values

in Å) between molecular portions of mefloquine (in the same orientation shown in Figure 4) and amino acid groups (right).

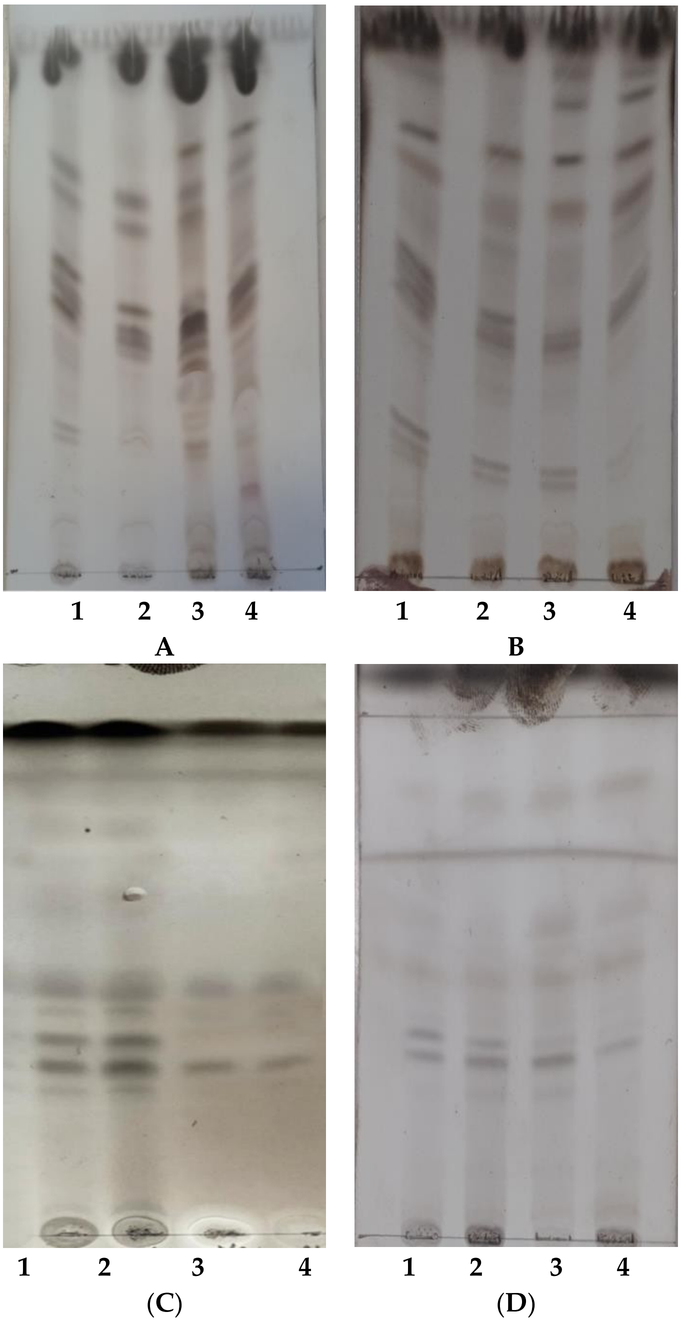

Figure S3. Effects of the different concentrations of compounds on *Mab* cell wall composition. *Mab* cells were grown for 48 hours in the absence/presence of compounds, then lipid composition was analyzed as in Material and Methods. **A**: TLC analysis of the complete lipid profile of cells grown in the presence of NSC135792. **B**: TLC analysis of the complete lipid profile of cells grown in the presence of mefloquine. **C**: total mycolic acid methyl esters (MAMEs) and fatty acid methyl esters (FAME) extracted from delipidated cell pellets of cultures grown in the presence of NSC135792. **D**: total MAMEs and FAME extracted from cultures grown in the pres-ence of mefloquine. Lane 1: no addition; lane 2: 1X MIC; lane 3: 10X MIC; lane 4: 25X MIC (4).
